# Apical CFTR Expression in Human Nasal Epithelium Correlates with Lung Disease in Cystic Fibrosis

**DOI:** 10.1371/journal.pone.0057617

**Published:** 2013-03-06

**Authors:** Marit Arianne van Meegen, Suzanne Willemina Julia Terheggen-Lagro, Kirsten Judith Koymans, Cornelis Korstiaan van der Ent, Jeffrey Matthijn Beekman

**Affiliations:** 1 Department of Pediatric Pulmonology, University Medical Center Utrecht, Utrecht, The Netherlands; 2 Centre for Molecular and Cellular Intervention, University Medical Center Utrecht, Utrecht, The Netherlands; 3 Department of Immunology, University Medical Center Utrecht, Utrecht, The Netherlands; University of Tübingen, Germany

## Abstract

**Introduction:**

Although most individuals with cystic fibrosis (CF) develop progressive obstructive lung disease, disease severity is highly variable, even for individuals with similar *CFTR* mutations. Measurements of chloride transport as expression of CFTR function in nasal epithelial cells correlate with pulmonary function and suggest that F508del-CFTR is expressed at the apical membrane. However, an association between quantitative apical CFTR expression in nasal epithelium and CF disease severity is still missing.

**Methods and Materials:**

Nasal epithelial cells from healthy individuals and individuals with CF between 12–18 years were obtained by nasal brushing. Apical CFTR expression was measured by confocal microscopy using CFTR mAb 596. Expression was compared between both groups and expression in CF nasal epithelial cells was associated with standardized pulmonary function (FEV_1_%).

**Results:**

The proportion of cells expressing apical CFTR in columnar epithelium is lower in CF compared to non-CF. The apical CFTR expression level was significantly correlated with FEV_1_% in F508del homozygous subjects (r = 0.63, p = 0.012).

**Conclusion:**

CFTR expression in nasal epithelial cells is lower in subjects with CF compared to healthy subjects. The proportion of cells expressing F508del-CFTR at the apical membrane is variable between subjects and is positively correlated with FEV_1_% in F508del-CFTR homozygous subjects.

## Introduction

Cystic fibrosis (CF) is caused by mutations of the *CFTR* gene that encodes a chloride channel predominantly expressed at the apical membrane of epithelial cells [1]. Over 1800 mutations in the CFTR gene have been described (www.genet.sickkids.on.ca/www.CFTR2.org) which are classified into severe and mild mutations based on functional activity in *in vitro* cell models and on association with clinical disease [2], [3]. The most common mutation contains a deletion of a phenylalanine at position 508 (F508del) that leads to incorrect folding, retention in the endoplasmic reticulum (ER) and degradation of the CFTR protein [4], [5].

Pulmonary failure is the leading cause for mortality, however, pulmonary morbidity is highly variable, even between individuals that share similar *CFTR* mutations [5]. Besides the *CFTR* gene other factors such as environmental factors and modifier genes have been indicated by twin studies to contribute significantly to CF phenotype [6]. These modifier genes could indirectly affect CF lung disease as has been shown for e.g. polymorphisms in the *TGFβ1* gene [7], but could also directly affect mutant CFTR protein function [8]. Bronsveld *et al*. and Thomas *et al.* observed differences in CFTR-mediated chloride transport between subjects homozygous for the F508del mutation by nasal potential difference (NPD) measurements that were positively correlated to pulmonary function [8], [9]. These data suggest that individual factors may directly modify residual function of F508del-CFTR. However, an association between apical CFTR expression in nasal epithelium and CF disease severity is still missing.

Nasal epithelium has been used to study CF disease severity as a low-invasive substitute for lung epithelium [8], [10], [11]. Contrasting data have been published on apical CFTR expression levels in nasal epithelial CF cells from homozygous F508del-CFTR subjects [12]–[16]. Lower apical CFTR expression levels were not only determined by CF status, but can also be affected by cell morphology, cell dedifferentiation and airway remodeling [17], [18].

Here, we investigated apical CFTR localization of nasal epithelial cells obtained from subjects with CF and healthy controls, and assessed associations with lung disease. Since the nasal cavity consists of heterogeneous populations of cells that differentially express CFTR that are all collected by nasal brushing, we used E-cadherin to select for epithelial cells, and used Ezrin as marker for apical membranes as previously performed by Kalin *et al*. [14], [19]. This allowed us to analyze CFTR in columnar epithelial cells with intact apical membranes that were not disturbed by the isolation procedure. We detected apical CFTR expression in CF cells although the proportion of cells expressing CFTR at the apical membrane was lower compared to non-CF cells. Furthermore, we observed that the proportion of cells expressing F508del-CFTR at the apical membrane is variable between subjects and is positively associated with FEV_1_%. These data suggest that CF disease severity in genotype-identical CF subjects could be due to apical CFTR expression.

## Materials and Methods

### Study Subjects

This study was conducted according to the principles expressed in the Declaration of Helsinki. The Study was approved by the Medical Research Ethics Committee of the UMC Utrecht, Ref 10–095. Individuals with CF were recruited from the Cystic Fibrosis Center of the University Medical Center Utrecht, the Netherlands. All individuals with CF (17; six male), aged 14.9±1.8 years, and their parents provided written informed consent for the collection of samples and subsequent analysis. Subjects with CF were eligible for the study if they were between 12–18 years old and clinically stable meaning, no clinical symptoms of a current respiratory infection and stable FEV_1_% over the past year. Samples were collected at a regular three monthly out-patient visit. All subjects with CF were genotyped and categorized as homozygous F508del-CFTR, compound heterozygous for F508del-CFTR, or two other mutations (characteristics of the study group are presented in Table 1). Lung function was measured using the Zan 500 spirometry system (nSpire, Oberthulba, Germany). The forced expiratory volume in one second, expressed as percentage of predicted (age, sex, height) values (FEV_1_%) was assessed using the Quanjer reference data set [20]. Collection and staining of the nasal epithelial cells and lung function testing were performed on the same day.

**Table 1:** Characteristics of the study group.

Subject	Mutation	Sex	Age (years)	Fev1%	*P.aeruginosa*	Pancreas
**1**	F508del/F508del	M	16.	68%	yes	insufficient
**2**	F508del/F508del	M	18.	62%	yes	insufficient
**3**	F508del/A455E	F	18.	48%	yes	sufficient
**4**	F508del/S1251N	F	14.	74%	yes	insufficient
**5**	F508del/365insT	F	12.	93%	no	insufficient
**6**	F508del/F508del	F	17.	90%	yes	insufficient
**7**	F508del/R553X	F	14.	110%	no	insufficient
**8**	F508del/F508del	F	14.	90%	no	insufficient
**9**	F508del/F508del	M	16.	95%	yes	insufficient
**10**	F508del/IVS11-I G>C	M	12.	114%	no	insufficient
**11**	F508del/F508del	F	14.	105%	no	insufficient
**12**	F508del/4243-3T>A	M	12.	79%	no	sufficient
**13**	F508del/1717-1G>A	F	15.	92%	no	insufficient
**14**	F508del/F508del	F	13.	76%	no	insufficient
**15**	F508del/F508del	F	16.	76%	yes	insufficient
**16**	F508del/F508del	F	14.	88%	no	insufficient
**17**	F508del/F508del	M	14.	57%	yes	insufficient

Abbreviations: FEV1% Forced experatory volume in one second expressed as percentage of predicted,

P.aeruginosa; Pseudomonas aeruginosa.

Pancreatic insufficiency was based on elastase levels in stool and dependency on pancreatic enzyme replacement therapy.

*Pseudomonas aeruginosa* colonization was defined according to the modified Leeds criteria, (chronic infection is defined as >50% of the sputum samples positive, collected during the last 12 months with at least 4 sputum samples during that period) [21]. These subjects failed to respond to *Pseudomonas aeruginosa* eradication therapy, and are currently treated with anti-*Pseudomonas* therapy (combination of azithromycin with inhaled antibiotics).

Healthy control subjects were included from the clinical and laboratory staff. 17 (Nine male) healthy subjects, aged 30.8±6.4 years, without upper respiratory tract infection, ciliary dyskinesia, or allergic rhinitis, were included after informed consent was given. Two healthy control subjects (two male) that smoked were included. One control started smoking during the study period and we observed no difference in CFTR measurements in time for this control subject.

### Human Nasal Epithelial Cells

Nasal epithelial cells were collected from healthy volunteers or subjects with CF as described [19]. Cytological brushes were obtained from cell tip (Servoprax, Wesel, Germany). Cells were collected in cold DMEM and centrifuged at 800 × g for 5 min, resuspended in cold PBS with EDTA 5 mM for 15 min, and filtered through cup Filcons 50 µm (BD, San Jose, CA) to generate single cell suspensions.

### Primary Antibodies

The following CFTR-specific antibodies were used: mAb L12B4 (mIgG2a, Chemicon international; Temecula, CA) and mAb 596 (mIgG2b) raised against amino acid residues 1204–1211 obtained via the Cystic Fibrosis Foundation Therapeutics (www.cftrfolding.org/CFFTReagents.htm) [22]. Other antibodies used in this study were mIgG2a anti-E-cadherin (BD) and mIgG1 anti-Ezrin (BD).

### Intracellular Staining and Confocal Microscopy

Intracellular staining of the nasal cells was performed as described [19]. Briefly, cells were fixed and permeabilized using Cytofix and Perm (BD) for 30 min at 4°C as were all following steps. Thereafter, cells were blocked with 10% goat serum (Jackson Immunoresearch, West Grove, PA) and subsequently incubated with mIgG2a anti-E-cadherin (6.25 µg/ml), mIgG1 anti-Ezrin (6.25 µg/ml) and mIgG2b anti-CFTR 596 (1∶250). The following cross-absorbed secondary antibodies were used; goat anti-mouse IgG1 Dylight 555-conjugated (4 µg/ml, Invitrogen, Carlsbad, CA, USA), goat anti-mouse IgG2a Dylight 488 and goat anti-mouse IgG2b Dylight 649 (both 1 µg/ml, Jackson Immuno Research). Cells were mounted in mowiol containing DAPI (4′,6-diamidino-2-phenylindole, Sigma, Zwijndrecht, Netherlands) and covered with glass coverslips. Tall columnar E-cadherin and Ezrin positive epithelial cells were selected by ocular and CFTR expression was then acquired in a blinded fashion upon laser scanning using a Zeiss LSM710 confocal microscope with a 63 × objective and oil immersion (Zeiss, Heidelberg, Germany).

### Statistical Analysis

Samples containing less than seven cells were excluded, and on average 20 cells were analyzed per sample. Four observers scored all confocal microcopy images twice in a blinded fashion. Intraclass correlation coefficient (ICC) was used to evaluate intra-and inter-observer agreement. Differences between and within observers were plotted against the mean and the mean difference and the limits of agreement, as described by Bland and Altman [23]. Averages of all the scores from one sample were used for statistical analysis. Kolmogorov-Smirnov test was used to test whether the variables were normally distributed. Unpaired Student’s t-test was used to compare normally distributed data, which were expressed as mean ± standard error of the mean (SEM). Correlations between FEV_1_% and CFTR expression were assessed using Pearson correlation. All data were analyzed in IBM SPSS Statistics 20.0 for Windows (IBM Corp, Armonk, NY). Statistical significance was accepted at a p-value less than 0.05.

## Results

### Immunolocalization of CFTR in Nasal Columnar Epithelial Cells

**Figure 1:**
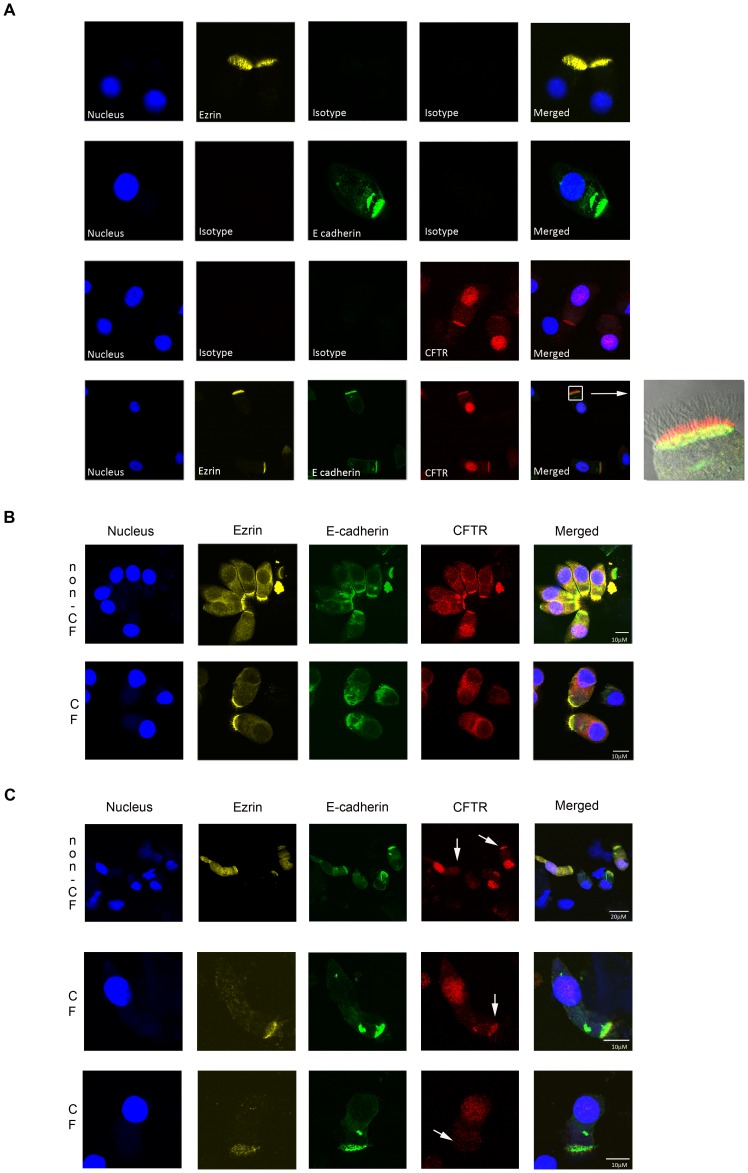
Immunolocalization of CFTR in nasal epithelial cells by confocal microscopy. Nasal cells were collected on ice, enriched for single cells, fixed and stained for Ezrin (yellow), E-cadherin (green), and CFTR (red). Nuclei are indicated by DAPI (blue). **A.** Specificity of the antibodies was assessed by comparison with corresponding isotype controls. Differential interference contrast image was included in the last merged picture. **B.** Representative examples of non-CF and CF columnar epithelial cells with similar Ezrin and E-cadherin staining. **C.** Representative examples of variability in apical CFTR expression within columnar epithelial cells from one individual. Upper panel; cells obtained from a non-CF individual. Lower two panels; cells obtained from an individual with CF (F508del/F508del). White arrow points to apical membranes with or without CFTR.

The nasal epithelium consists of a heterogeneous population of cells including ciliated or columnar epithelial cells that express CFTR at the apical surface, and non-CFTR expressing cells such as goblet and basal cells [15]. We used E-cadherin to mark epithelial columnar cells and Ezrin to detect intact apical regions. We verified specificity of the antibodies used in this study by comparison with isotype-matched control antibodies (Fig. 1A). We found no clear differences between Ezrin and E-cadherin localization between CF cells and non-CF cells (Fig. 1B). Ezrin and E-cadherin positive cells with intact apical villi were selected to analyze CFTR expression. To prevent selection bias for CFTR expression, we deliberately analyzed CFTR in a fluorescent channel that could only be visualized after laser scanning, and was thus not visible when selecting for Ezrin and E-cadherin positive cells. Murine mAb 596 was used to detect CFTR as we found that this mAb is optimally suited to detect F508del in ectopic expression settings by immunofluorescence [24]. We regularly observed some nuclear staining using mAb 596 that varied between subjects and within subjects that we interpreted as non-specific recognition (Fig. 1A and C), but also observed perinuclear staining that may represent ER-localized CFTR (Fig. 1B). In different cells within the same subject (either a healthy donor, or subject with CF), we observed clear differences in apical CFTR expression (representative examples are shown in Fig. 1C). These data indicated that a combination of Ezrin, E-cadherin, and CFTR mAbs could be used to specifically detect apical CFTR in columnar epithelial cells.

### Reproducibility of Apical CFTR Expression Measurement

To determine observer variation, four observers that were not involved in processing the samples scored the proportion of apical CFTR expressing cells within the Ezrin and E-cadherin positive cell population. All observers scored the same samples twice. The intraclass correlation coefficient (ICC) was found to be >0.97 for all observers (Table 2 shows an overview of measurements; Bland-Altman plots are presented in Figure S1). For the inter-observer variability the ICC score was also >0.97. Furthermore, the scores of the different observers showed a good level of agreement when assessed by Bland and Altman plots (Table 2).

**Table 2:** Intraclass correlation coefficient, mean differences and limits of agreement among observers.

	ICC	Meandifferences	Limits ofagreement
**Intra-observer variability**			
**observer 1**	0.975	3.33	−13.47 to 20.14
**observer 2**	0.990	−3.97	−14.40 to 6.57
**observer 3**	0.973	2.33	−15.20 to 19.87
**observer 4**	0.985	1.057	−11.70 to 13.87
**Inter-observer variability**			
**observer 1 vs. 2**	0.999	−1.042	−4.905 to 2.821
**observer 1 vs. 3**	0.989	−5.500	−16.68 to 5.682
**observer 1 vs. 4**	0.980	−2.079	−16.69 to 12.53
**observer 2 vs. 3**	0.985	−4.458	−17.16 to 8.247
**observer 2 vs. 4**	0.979	−1.038	−16.24 to 14.17
**observer 3 vs. 4**	0.988	3.421	−7.914 to 14.75

Abbreviations: ICC; Intraclass correlation coefficient.

**Figure 2:**
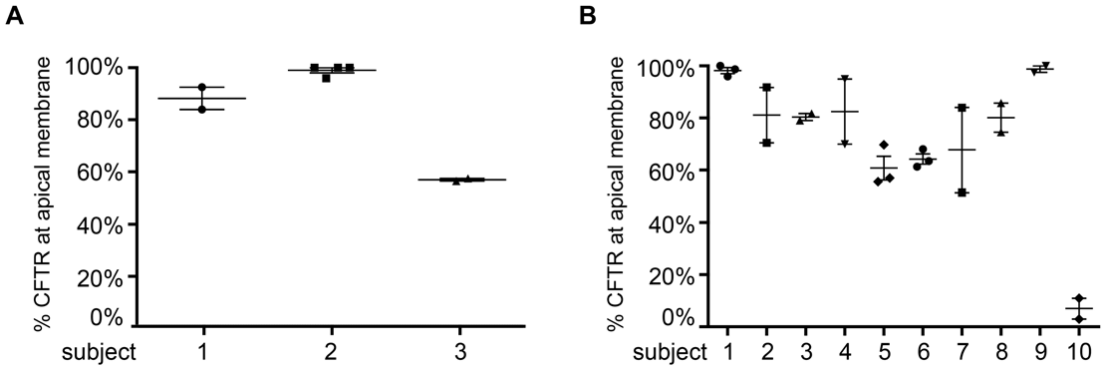
Analysis of apical CFTR expression in nasal columnar epithelial cells. The percentage of cells expressing CFTR at the apical membrane was analyzed within the E- cadherin and Ezrin positive cell compartment. Per sample on average 20 cells were counted. **A.** Technical variability. For three subjects, staining was performed in duplicate or quadruplicate. Proportion of columnar epithelial cells expressing apical CFTR was compared between the duplicates. Number one represents a subject with CF. **B.** Biological variation. 10 individuals were brushed several times and apical CFTR expression in columnar epithelial cells was compared between the different time points for each individual. Number ten represents a subject with CF.

Technical variability of the detection procedure was low as indicated by analysis of multiple duplicate or quadruplicate samples from a single brushing (Fig. 2A). Next, we assessed intra-subject variability in apical CFTR expression by analysis of several individuals (CF or healthy) at various time intervals ranging between three weeks and six months (Fig. 2B). For most individuals, apical CFTR expression levels were identical, however, we observed limited variation for some individuals that either reflects biological modulation of CFTR or technical variability. To conclude, apical CFTR expression in nasal epithelium can be analyzed by confocal microscopy in a single-blind setting, with low observer bias and technical variation, and was reproducible in most subjects over time.

### Apical CFTR Expression in Nasal Epithelial Cells of Healthy and CF Subjects

**Figure 3:**
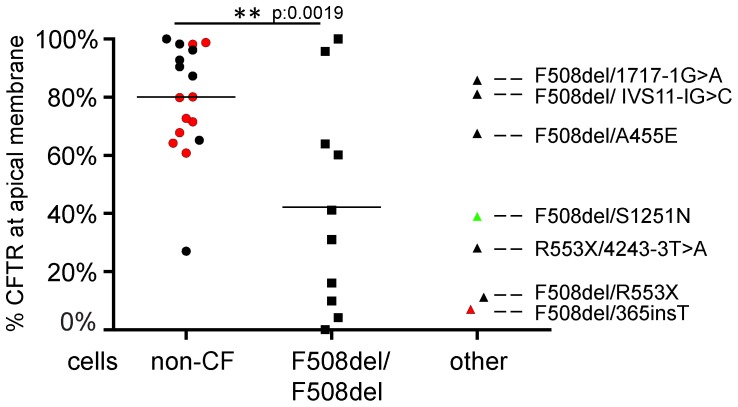
Apical CFTR expression in non-CF cells compared to CF cells. Ezrin and E-cadherin positive cells were analyzed for apical CFTR expression per individual by all observers and the mean percentage of cells displaying apical CFTR was plotted. **A.** Differences in distribution for the proportion of columnar epithelial cells expressing apical CFTR in non-CF individuals (n = 17) compared to individuals with CF homozygous for F508del-CFTR using Student’s t-test. Apical CFTR expression levels for CF patients with other mutations are displayed at the right scatter plot. A subject that expresses a CFTR allele associated with reduced gating is indicated in green (F508del/S1251N). Repetitive measurements of a single subject are indicated in red.

Next, we examined apical CFTR expression in columnar nasal epithelial cells obtained from 17 non-CF individuals and 17 individuals with CF (Fig. 3). Several individuals were brushed at various times as described in Figure 2B and their average values determined by all observers, are presented. Approximately 80% of the columnar nasal epithelial cells obtained from non-CF individuals showed apical CFTR expression. For all subjects with CF, we observed an average of 40% of the cells expressing apical CFTR, indicating apical CFTR expression is lower in CF as compared to healthy controls (p = 0.001; 95% CI 0.16–0.55). These results were verified with another CFTR-specific mAb L12B4 that demonstrated average apical expression levels in non-CF cells of 81% and in CF cells of 45% (non-CF ten individuals, eight individuals with CF; data not shown). Subsequently, we studied apical CFTR expression within CF subgroups and observed apical CFTR levels ranging between 0–100% for the F508del-CFTR homozygous subjects (Fig. 3).

High and low apical CFTR expression was observed in F508del-CFTR compound heterozygous patients (see Table 3 for classification of mutations) that carried class I mutations as second allele (1717-1G>A and R553X, respectively), suggesting that mono-allelic expression of F508del-CFTR could be sufficient to drive high levels of apical expression (Fig. 3, right scatter plots). Apical expression in a subject with S1251N was somewhat lower than anticipated since we expected that CFTR mutations that affect gating or conductivity would be expressed at levels of healthy control subjects. In conclusion, the proportion of nasal epithelial cells that apically express CFTR is variable for both non-CF and CF individuals, and ranged between 0–100% for homozygous F508del-CF individuals.

**Table 3:** Classification of CFTR mutations in this study.

1717-1G>A	Class I	altered splicing
365insT	Class I	frameshift
4243-3T>A	Class V	altered splicing
A455E	Class II, V	missense
F508del	Class II, III, VI	amino acid deletion
IVS11-1G>C	Class I	altered splicing
R553X	Class I	nonsense
S1252N	Class III	missense

### Apical CFTR Expression in Nasal Epithelial Cells Correlates with Pulmonary Function

**Figure 4:**
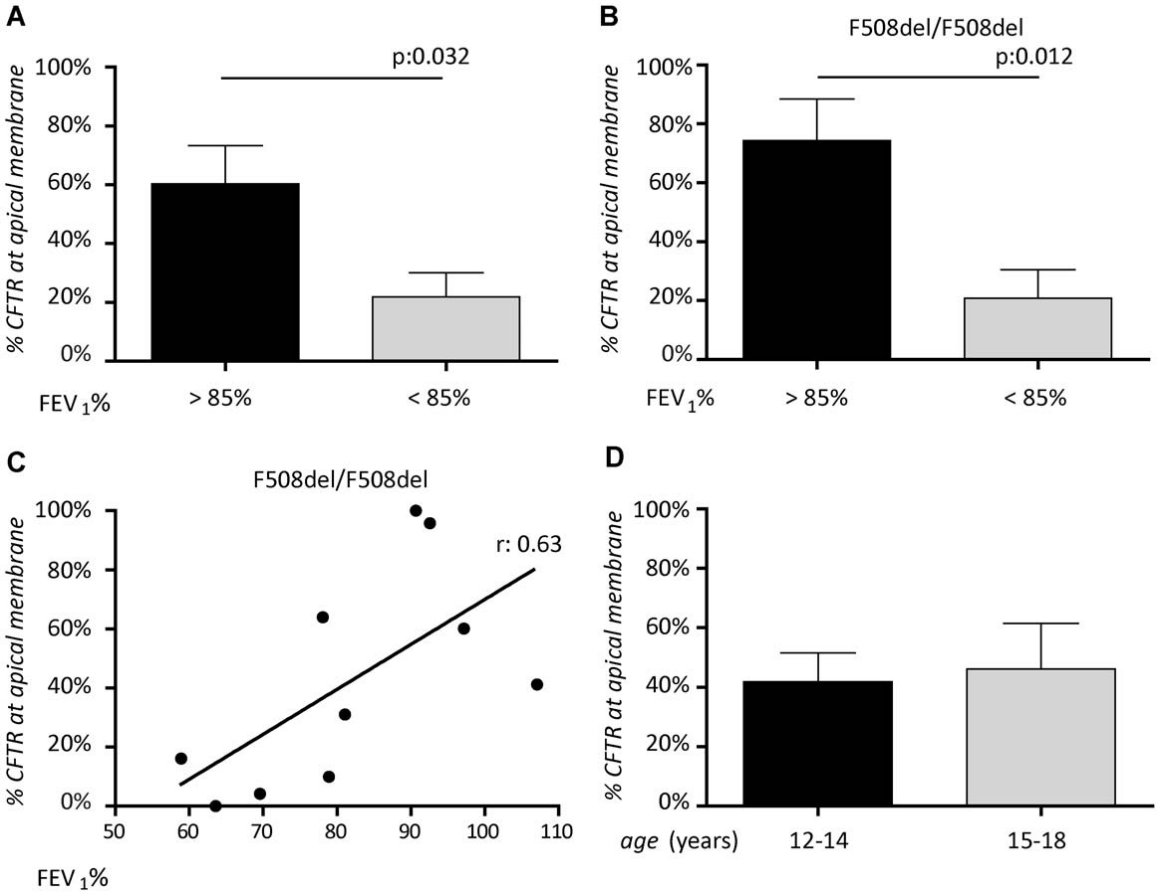
Correlation between apical CFTR expression in nasal epithelial cells and clinical parameters. **A. and B.** Apical CFTR expression levels were determined in subjects with CF with FEV_1_ above 85% to FEV_1_ below 85%. Linear regression was used to analyze the association between apical CFTR expression and FEV_1_% for individuals homozygous for F508del mutation. The correlation coefficient is indicated. **D.** Apical CFTR expression levels were compared between age groups 12–14 years and 15–18 years for all subjects with CF. Statistical analysis was performed using Student’s t-test.

Since differences in the proportion of columnar cells expressing apical CFTR were observed within the individuals with CF, we assessed if apical CFTR expression could be related to CF disease phenotype. First, we studied the relation between apical CFTR expression and pulmonary function indicated by percent predicted forced expiratory volume in one second (FEV_1_%). When all subjects with CF were included, subjects with high FEV_1_% appeared to display higher apical CFTR expression levels, which almost reached statistical difference in this limited cohort (p = 0.054, Fig. 4A). A similar trend was observed when the subject containing F508del/S1251N that contained a gating mutant that can be expected to be expressed at levels of wt-CFTR, was excluded from analysis. In F508del-CFTR homozygous subjects, we observed a strong and statistically significant correlation between lung function and apical CFTR (r 0.63; p = 0.012, 95% CI 0.15–0.92; Fig. 4B and C). This correlation was independent of age (Fig. 4D). No correlations between apical CFTR expression level and *P. aeruginosa* infection was observed (Figure S2). In conclusion, there is a significant positive correlation between apical CFTR expression levels in columnar nasal epithelial cells obtained from F508del-CFTR homozygous subjects and pulmonary function.

## Discussion

In this study we described a reproducible method to quantify CFTR expression in nasal epithelial cells. We detected apical CFTR expression in non-CF cells as well as CF cells, and found large variability in the proportion of cells expressing CFTR at the apical membrane in F508del-CFTR homozygous subjects. Conflicting data has been published on the subcellular localization of F508del-CFTR in native airway tissues [12], [13], [16]. Our and other recent data support a model in which F508del-CFTR subcellular localization is regulated by patient-specific factors. Factors within the *CFTR*-gene such as revertant mutations that counteract the destabilizing effect of F508del on CFTR conformation [25], or factors outside the *CFTR-*gene that control F508del production, folding, trafficking and/or degradation could be different between subjects and modify apical CFTR expression.

The large differences in % apical CFTR expressing cells that we observed within the F508del homozygous subjects were found to positively correlate with pulmonary function. This correlation did decrease to borderline significance (p = 0.054) when subjects with other genotypes were included, likely due to small sample size. For subjects homozygous for F508del-CFTR, variation in residual CFTR activity has been described [8], [9], [11] that may correlate with apical CFTR expression. Other mechanisms responsible for the association between lung function and apical CFTR expression may include functional modulation of other apically located proteins such as epithelial sodium channel [26] or outwardly rectifying chloride channel [27] that depend on apical CFTR localization rather than CFTR function. Mutant apical CFTR may also play a role in pathogen recognition and clearance [28], [29]. A paired analysis of apical CFTR expression and nasal potential differences or intestinal current measurement could generate important insights into the relationship between apical CFTR expression, residual CFTR function or functional modulation of other ion channels, and lung function.

An important strength of this study was that CFTR expression was selectively analyzed in E-cadherin positive columnar epithelial cells with intact apical regions as indicated by Ezrin [15], [30]. In addition, we used Ezrin because it interacts with CFTR through PDZ scaffolding protein Na^+^/H^+^-exchanger regulatory factor (NHERF)/ezrin-radixin-moesin binding phosphoprotein-50 (EBP-50) [31]–[33] and modulates CFTR activity [34]. No differences were observed in staining pattern for E-cadherin and Ezrin between non-CF and CF cells (Fig. 1B), which is in agreement with Kreda *et al.* [15].

Our studies were performed using a specific CFTR mAb (596) based on its capacity to stain apical CFTR in brushed nasal cells, its ability to detect F508del-CFTR by immunofluorescence in ectopic expression models [24], and because it is phosphorylation insensitive [19], [35]. Apical CFTR detection by mAb 596 is likely specific as similar fractions of % apical CFTR expressing cells in healthy controls and subjects with CF were obtained using a second CFTR specific mAb (L12B4), and no apical signal was observed using isotype matched control mAbs. However, we were not able to include class I *CFTR* null allele homozygous subjects to formally proof that apical staining was specific. We also found that mAb 596 reacted with nuclear antigens (see Fig. 1A and C), which was variable between and within donors (independent of *CFTR* genotype) and hampered analysis of apical CFTR levels relative to total CFTR per cell. The strict selection of columnar cells with intact apical regions, and the use of mAb 596 may have been important to demonstrate the relation with pulmonary function that we observe here in this study.

We further validated our measurement by using multiple blinded observers and technical duplicates, and showed that analysis of the proportion of cells expressing apical CFTR is robust when sufficient cell numbers can be analyzed. We observed minimal biological variation in apical CFTR expression over time using subjects that displayed apical CFTR expression levels dispersed over the dynamic range of the measurements. Variation in apical expression may be related to biological processes interfering with CFTR expression. Inflammatory cytokines such as TNFα can directly affect CFTR mRNA levels [18], and β_2_- adrenergic receptor stimuli have been found to modulate interactions between CFTR and apical membrane components [36]. In addition, sex hormones could also affect CFTR expression as suggested by Fanelli *et al*., and Sweezey *et al.* [37], [38].

For our analysis, subjects with CF between 12–18 years were included as pulmonary function starts to decline within this age group (Figure S3). Pulmonary function was indicated by percent predicted forced expiratory volume in one second (FEV_1_%) adjusted for age, height and gender to limit the impact of these confounding factors. We used an international reference data set [20] and results were comparable with our own reference data set for Dutch children [39]. We compared our CF data set with adult healthy subjects due to ethical considerations that may potentially introduce an age-bias when comparing healthy and CF subjects. However, this appears limited since we observed no significant relation between apical CFTR expression and age in the healthy control group.

Ideally, a greater number of cells per subject would have been analyzed, on average this was 20 cells per individual. This was limited due to extensive reprocessing of the samples as result of the E-cadherin and Ezrin inclusion criteria and variability of the brushing procedure itself. Individual factors modifying the yield of brushed cells appeared to include oxygen supply and nasal gastric tube at night, probably due to direct damage of the tubes to the epithelial airway layer. However, biological variation was minimal also for the samples with low cell numbers.

To our knowledge, this is the first report describing a relation between apical CFTR expression in nasal columnar epithelial cells and lung disease in homozygous F508del-CFTR subjects. These data require verification in larger cohorts, and in longitudinal studies to establish the predictive values of apical CFTR expression for lung disease. The considerable variation in apical CFTR expression levels in F508del-CFTR homozygous subjects may also be linked to responses on CFTR-directed therapy as was previously shown for gentamycin or Ataluren-induced CFTR correction in subjects with CF with a nonsense mutation [40], [41]. Also, F508del-CFTR homozygous subjects with high levels of apical CFTR may benefit directly from potentiator monotreatment as has been observed [42]. Differences in apical CFTR expression between patients may be used to tailor personalized medicine approaches for CFTR-restoring drugs in the future, and may select individuals that are at increased risk to develop lung disease.

## Supporting Information

Figure S1 **Bland-Altman plots.** Graphic representation of the intra-observer variability for four observers. The broken line represents the mean difference, the dotted lines represent 95% limits of agreement. A–D observer 1–4.
(TIF)

Figure S2 **Correlation between apical CFTR expression in nasal epithelial cells and** ***P. aeruginosa*** **infection.** Apical CFTR expression levels were compared between the individuals with CF with chronic *P. aeruginosa* infection and without infection.
(TIF)

Figure S3 **Correlation between age and standardized pulmonary function.** Correlation between age and FEV_1_% for individuals homozygous for F508del mutation.
(TIF)
