# Supplementary figures and images for: Apical CFTR Expression in Human Nasal Epithelium Correlates with Lung Disease in Cystic Fibrosis
Source: PLoS One. 2013 Mar 6;8(3):e57617. doi: 10.1371/journal.pone.0057617 (PMC3590182; doi:10.1371/journal.pone.0057617)

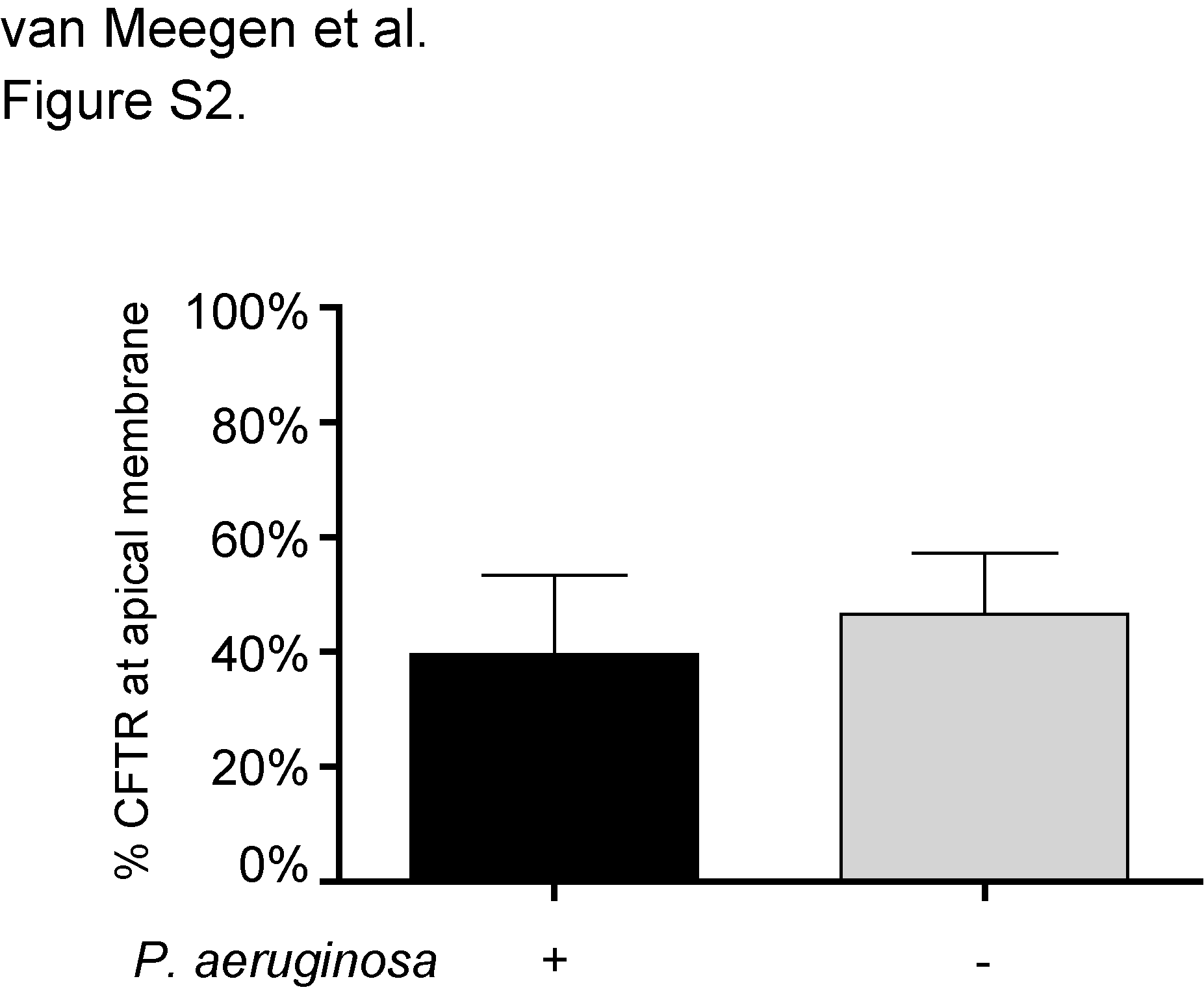

Supplement: Figure S2 — Correlation between apical CFTR expression in nasal epithelial cells and P. aeruginosa infection. Apical CFTR expression levels were compared between the individuals with CF with chronic P. aeruginosa infection and without infection. (TIF) [file pone.0057617.s002.tif]

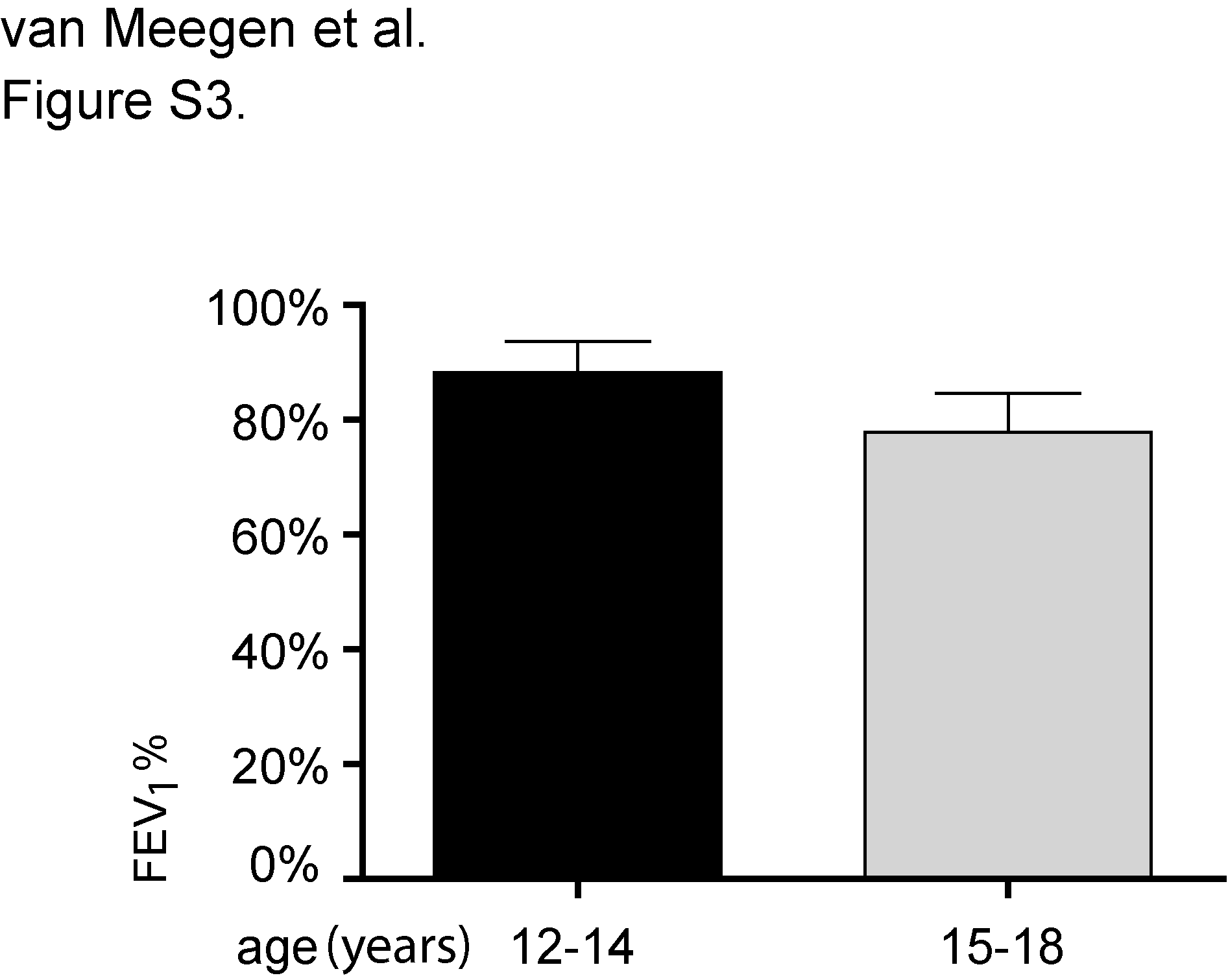

Supplement: Figure S3 — Correlation between age and standardized pulmonary function. Correlation between age and FEV1% for individuals homozygous for F508del mutation. (TIF) [file pone.0057617.s003.tif]
